# Language as Description, Indication, and Depiction

**DOI:** 10.3389/fpsyg.2018.00716

**Published:** 2018-05-23

**Authors:** Lindsay Ferrara, Gabrielle Hodge

**Affiliations:** ^1^Department of Language and Literature, Norwegian University of Science and Technology, Trondheim, Norway; ^2^Deafness Cognition and Language Centre, University College London, London, United Kingdom

**Keywords:** sign language, multimodal, semiotics, language, indexicality, depiction

## Abstract

Signers and speakers coordinate a broad range of intentionally expressive actions within the spatiotemporal context of their face-to-face interactions (Parmentier, 1994; Clark, 1996; Johnston, 1996; Kendon, 2004). Varied semiotic repertoires combine in different ways, the details of which are rooted in the interactions occurring in a specific time and place (Goodwin, 2000; Kusters et al., 2017). However, intense focus in linguistics on conventionalized symbolic form/meaning pairings (especially those which are arbitrary) has obscured the importance of other semiotics in face-to-face communication. A consequence is that the communicative practices resulting from diverse ways of being (e.g., deaf, hearing) are not easily united into a global theoretical framework. Here we promote a theory of language that accounts for how diverse humans coordinate their semiotic repertoires in face-to-face communication, bringing together evidence from anthropology, semiotics, gesture studies and linguistics. Our aim is to facilitate direct comparison of different communicative ecologies. We build on Clark’s (1996) theory of language use as ‘actioned’ via three methods of signaling: describing, indicating, and depicting. Each method is fundamentally different to the other, and they can be used alone or in combination with others during the joint creation of multimodal ‘composite utterances’ (Enfield, 2009). We argue that a theory of language must be able to account for all three methods of signaling as they manifest within and across composite utterances. From this perspective, language—and not only language use—can be viewed as intentionally communicative action involving the specific range of semiotic resources available in situated human interactions.

## Introduction

How do humans communicate with each other? One might say there are many paths up the mountain: a hearing speaker describes the use of a basket fish trap by closely aligning his speech with manual gestures depicting the shape of the trap and how it functions (Enfield, 2009, p. 188); a deaf signer unifies lexicalized manual signs within a bodily re-enactment of herself as a young child to express the sense of surprise and wonder she experienced as she learned signed language for the first time (Fenlon et al., 2018, p. 96); while a deafblind signer reaches for the hand of a hearing shopkeeper, gestures “how much?”, and then invites the shopkeeper to trace numbers on his palm (Kusters, 2017, p. 400). In each context, each individual engages with others in their environment on their own terms, making use of the various bodily articulators (a voice, hands, body) and strategies for communicating (speech, visible and tactile actions, numerical symbols) available to them in that moment and physical space. In doing so, they position themselves as independent agents embedded within an intricate and dynamic network of social relationships, someone who effects social actions and is affected by others’ actions in turn (Levinson and Enfield, 2006; Enfield, 2013).

Despite the sheer variety of communicative practices that can be observed, many linguists have historically been interested in the question of how ‘language’ – defined as symbolic, conventionalized, and paradigmatic arrangements for making meaning – works. This has typically involved analyzing communicative phenomena using a Saussure-inspired semiological approach in which the linguistic *signe* is viewed as a dual entity of ‘signifier’ and ‘signified.’ The focus has therefore been on those symbolic and conventional pairings of form and meaning that are componential (e.g., phonology, morphosyntax) and therefore easier to identify and analyze. Within this paradigm, the arbitrariness of symbolic *signe* relationships and their potentially decontextualized semantic power is emphasized, while the contextual rootedness and emergent meaningfulness of semiosis (namely, indexicality and iconicity) is often omitted (Parmentier, 1994, p. 5). Yet the aspects of language use which can be analyzed from a structuralist perspective are only part of the picture of how we engage in social actions and communicate: they do not explain everything.

While useful for understanding unimodal patterns of language use, such as the constituency-based analysis of speech or writing, these conventional symbol-driven approaches have resulted in theories of language that do not fully consider the semiotic plurality of human communication, nor how this plurality interacts with the emergence of such conventional symbols. Many researchers have challenged this narrow view of language and have shown how multimodal approaches to language description are necessary for a holistic understanding of human communication. For example, researchers from the field of gesture studies have investigated how to classify and analyze different types of co-speech gestures (e.g., McNeill, 1992; Goldin-Meadow, 2003; Streeck, 2009), including the identification of different types of gestures with respect to their function and degrees of conventionalization and grammaticalization (e.g., Kendon, 2004; Wilcox, 2007; Calbris, 2011; for an overview see Müller et al., 2013, 2014, especially Bressem, 2013). Signed language linguists have investigated the coordination of different types of signs and strategies for making meaning used by deaf signers (e.g., Sutton-Spence and Woll, 1999; Liddell, 2003; Johnston, 2012; Vigliocco et al., 2014), including recent efforts to directly compare the communication of deaf signers with hearing co-speech gesture (see Perniss et al., 2015, *inter alia*).

However, there has yet to be a general theory that unifies this evidence to account for diverse communicative practices. Furthermore, many researchers continue to work within paradigms that posit boundaries between ‘language’ and ‘gesture,’ ‘linguistic and ‘non-linguistic,’ ‘verbal,’ and ‘non-verbal’ (see Kendon, 2014). However, as Kendon (2014, p. 3) has argued, “we must go beyond the issue of trying to set a boundary between ‘language’ and ‘non-language,’ and occupy ourselves, rather, with an approach that seeks to distinguish these different systems, at the same time analyzing their interrelations.” How else can we directly and systematically compare the communicative practices used by the hearing fisherman and his interactant with those used by the deaf signer and her friend, or the deafblind signer and the shopkeeper? If elements of some repertoires are excluded, our understanding of the complex nature of language variation and diversity cannot progress. Our approach is rather to seek an understanding of how diverse humans (e.g., hearing, deaf) communicate using the semiotic repertoires available to them, and how the resulting conventions of these ecologies can be described empirically. To do this, we build upon Clark’s (1996) theory of language use as ‘actioned’ via three methods of signaling: describing, indicating, and depicting. These methods differ fundamentally in how they signify referents, yet each can be used alone or in combination with others during the joint creation of multimodal ‘composite utterances’ to effect social actions (Enfield, 2009).

We use Clark’s (1996) theory as a starting point, because it is based upon the foundational semiotic principles of ‘symbols, indices and icons’ first proposed by Peirce (1955). While other linguists and gesture researchers have also advocated Peircean-inspired semiotic approaches for analyzing multimodal language data (e.g., Mittelberg, 2008; Fricke, 2014) – and these approaches are certainly complementary to the one described here – we believe that Clark’s theory most clearly marries Kendon’s call for a ‘comparative semiotics’ of signed and spoken communication (Kendon, 2008) with existing semiotic approaches adopted by signed and spoken language linguists (e.g., Liddell, 2003; Enfield, 2009; Dingemanse, 2011; Johnston, 2013b). In taking a semiotic approach (rather than a formal linguistic or gesture-oriented one), we also strive for a modality-free understanding of the function and use of different semiotic acts, and therefore avoid issues that have arisen in approaches which do not consider more gradient aspects of meaning (see Okrent, 2002; Liddell, 2003). In the following sections, we review the literature on communicative practices and semiotic repertoires from an ecological perspective (Haugen, 1972; Goodwin, 2000). We describe Clark’s three methods of signaling and the notion of the composite utterance (Enfield, 2009). We then bring together evidence from existing signed and spoken language research, and present examples of composite utterances from deaf signers and hearing speakers. All examples are reflective of the everyday practices signers and/or speakers use to describe, indicate, and/or depict meaning during their interactions. Finally, we conclude with some thoughts on re-orienting language theory to account for these varied communicative practices—thereby underscoring that a theory of language use should not be fundamentally different from a theory of language.

## Communication Practices and Semiotic Repertoires

The first step in investigating the communication practices of diverse humans is to consider the communicative ecologies in which these practices emerge. Signers and speakers live in richly dynamic communicative ecologies, in which what we understand as ‘language’ is just one of many resources available for making meaning (see Bühler, 1990/1934; Parmentier, 1994; Enfield, 2009; Keevallik, 2018). We coordinate varied bodily articulators (a voice, hands, body) and physical artifacts (e.g., paper, sand, mobile phone) to express communicative intent, the details of which are embedded within interactions occurring in a specific time and place. For example, in the Western desert region of Australia, Ngaanyatjarra children may incorporate alphabetic symbols into their stories drawn in the sand, along with the more traditional iconographic drawings and objects used by adults to index and depict referents in these stories. This youth-driven contribution to established sand story practices reflects generational literacy differences (Kral and Ellis, 2008; see also Green, 2014). Shared semiotic resources and modes of communication within ecologies may therefore be used in different ways by different individuals at different times.

In this sense then, a communicative ecology is not simply the environment in which signers and speakers act; it is the constantly emerging complex shape and history of interactions between language users and their environment (Haugen, 1972; Goodwin, 2000). These reciprocal, dynamic interactions give rise to ‘structural couplings’ (Maturana and Varela, 1987) between individuals and their environment, which manifest as varied communication practices. These practices evolve as signers and speakers draw on all meaningful resources available to them into a complete, heteroglossic package, i.e., the “semiotic repertoire” (Kusters et al., 2017). Within this cognitive/biosemiotics approach, a key principle is that the meanings which emerge within ecologies are largely inferential – more so than symbolic – so that tokens of expression stand in relation to each other with respect to their specific indexical properties (Peirce, 1955; see Kravchenko, 2006).

Another, closely-related principle is that the communication practices which emerge are embedded in the physical environment in which they occur (Duranti and Goodwin, 1992; Goodwin, 2000; Keevallik, 2018). This leads to the emergence of “spatial repertoires” which are defined by the communicative resources available to interactants in a particular place (Nevile et al., 2014; Pennycook and Otsuji, 2014). Encounters between agents in an ecology are developed and maintained over various time frames, with the effect that “future interactions occur in a new and adaptive way” (Pickering, 1997, p. 192). Small-scale social encounters between individuals shape larger scale practices and vice versa (Agha, 2005, p. 12). Consequently, communicative practices and repertoires share similarities and differences, both within specific interactions and across social networks, depending on where they unfold (Bourdieu, 1991; Agha, 2007; see also Bernstein, 2003/1971).

Diverse semiotic resources and modes of communication are used to disambiguate the situated context, whereby disambiguation is negotiated between interactants during social interactions via ostensive and inferential acts (LaPolla, 2003, 2005). These notions challenge generative understandings of situated context as being used to disambiguate fixed symbolic forms, whereby the interpretation of ostensive-inferential communication involves a coding-decoding process (cf. Sperber and Wilson, 1986; Wilson and Sperber, 1993). However, it is important to note that an individual’s repertoire is as much determined by the resources they do not have, as by those they do have (Busch, 2015, p. 14). This factor gains prominence, for example, during interactions between signers and/or speakers whose repertoires do not fully align, as they must actively negotiate which bits of each other’s repertoire can be used effectively – or not (see e.g., Green, 2015; Harrelson, 2017; Hodge, forthcoming). Crucially, an acknowledgment of semiotic diversity enables investigations of signed and spoken languages to relax from the restraints of ‘structure’ and ‘descriptive representation’ resulting from the lineage of de Saussure’s important contributions to linguistics. It re-establishes semiotic diversity as a foundation upon which to identify and explore patterns of embodied communication, of which conventionalized descriptive signaling is just one method, as we will see in the following sections.

## *P*-Signs Signaled Through Description, Indication, and Depiction

The emergence of diverse communicative practices can be at least partly attributed to the quintessentially face-to-face and multimodal nature of human interactions (Bavelas et al., 1997; Kelly, 2002, 2006; Kita, 2003; Tomasello, 2003; Cienki and Müller, 2008; Calbris, 2011; Müller et al., 2013, 2014). Indeed, the availability of space during face-to-face interactions between deaf signers has been suggested as “a fact that may influence, and even constrain, the linguistic [i.e., communicative] system in other ways” (Johnston, 1996, p. 1). These influences and constraints manifest in the extensive and habitual integration of tokens of three types of signs (in a Peircean sense) in face-to-face, situated discourse: (1) symbols, (2) indices, and (3) icons (Peirce, 1955; see also Parmentier, 1994; Kockelman, 2005; Mittelberg, 2008; Enfield, 2009; Fricke, 2014). Here we refer to tokens of these types of signs as ‘*P*-signs’ to avoid confusion with other uses of the term ‘sign.’ Clark (1996) proposed that symbols, indices, and icons are signaled through acts of describing, indicating and depicting.^1^ Language use is therefore a system of signaling with these three different methods.

**FIGURE 1:**
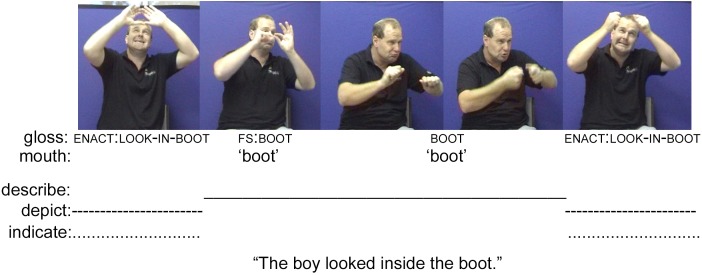
An example of a composite utterance in Auslan (images used with consent, Johnston, 2008).

**FIGURE 2:**
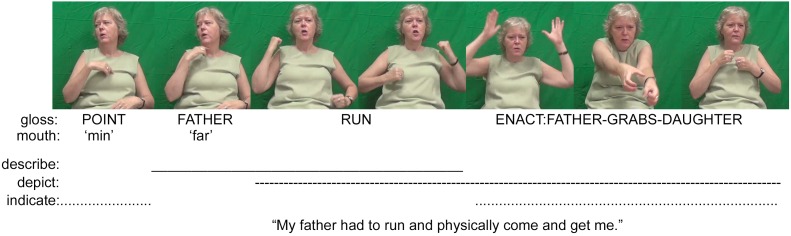
An example of a composite utterance in Norwegian Sign Language (images used with consent, Ferrara and Bø, 2015).

Symbols are form-meaning pairings where it is ‘pre-agreed’ that *X* stands for *Y*. Tokens of symbols are fully conventionalized and thus have both token and type identities (Enfield, 2009, p. 13). Examples of symbols include the lexicalized manual signs of deaf signed languages (e.g., the Auslan sign BOOT in **Figure 1** and the Norwegian Sign Language sign FATHER in **Figure 2**), alternate signed languages (see e.g., Kendon, 1988; Green, 2014), as well as the spoken or written words of spoken languages (e.g., the English words *booking a flight* in **Figure 5**). It also includes culturally-specific emblematic manual gestures such as the OK and THUMBS-UP gestures (see e.g., Sherzer, 1991), and even conventionalized intonation contours, such as in the English utterance “That was cold!” to mean cold-hearted (Liddell, 2003, pp. 358–361), or those whistled by Pirahã men when hunting (Everett, 2005).

Clark (1996) proposes that symbols are signaled through acts of description. It is these descriptions that have been the primary focus of linguistics. Dingemanse (2015) provides an apt characterization of descriptions:

*Descriptions are typically arbitrary, without a motivated link between form and meaning. They encode meaning using strings of symbols with conventional significations, as the letters in the word “pipe” or the words in a sentence like “the ball flew over the goal.” These symbols are discrete rather than gradient: small differences in form do not correspond to analogical differences in meaning. To interpret descriptions, we decode such strings of symbols according to a system of conventions* (Dingemanse, 2015, pp. 950–951).

It is true that understanding how description works is essential to language and linguistic theory. However, it is also true that actual utterances unfolding as parts of specific interactions and spatiotemporal contexts involve much more than description: utterances must index actual referents and meanings, and may therefore also include indices and depictions (Clark, 1996, pp. 161–162).

Indices are forms that anchor communicative events to a specific time and place. These forms are physically connected to their referents, e.g., through finger pointing, and work to create focused joint attention (Clark, 1996, pp. 164–165). Indices, as opposed to symbols, exhibit both conventional and non-conventional properties. Enfield (2009, p. 13) describes tokens of indices as partly-conventional symbolic indexicals that “[glue] things together, including words, gestures, and (imagined) things in the world.” These indexed referents may be physically present and jointly attended, or they may be entirely conceptual and mapped onto a jointly attended real space (Liddell, 1995). Indicating is therefore the method of signaling specific referents via indices using a variety of forms (Clark, 1996). For example, hearing speakers often signal indices using deictic forms such as the English function words *it* and *this*, as well as hand-pointing, lip-pointing, and other culturally-specific bodily actions during which speakers or signers extend parts of their body (or objects that act as an extension of their body) in a direction toward, or contacting, some referent in the context of the utterances (Clark, 1996; Kita, 2003; see also Fricke, 2014). The placement of material objects in a purposeful way in various settings is also a method of indicating (Clark, 2003).

The physical manifestation of pointing actions may also depend on whether agents within a given ecology preference signed or spoken modes of communication, as well as other constraints such as local, culturally-specific conventions and frequency of use. For example, analysis of pointing actions by speakers of Arrernte in Northern Australia has shown that the physical manifestation of these actions is culturally specific (not universal), with different forms potentially differentiating distinct frames of reference and semantic fields such as near vs. far proximity, absolute vs. relative space, and/or singular vs. plural entities (Wilkins, 2003). Corpus-based analysis of pointing actions produced by deaf native and near-native signers of Auslan (Australian signed language) from a semiotic perspective suggested that pointing actions in signed languages are not fundamentally different to the co-speech pointing actions produced by hearing speakers, and that the linguistic analysis of signed language pointing as fully grammaticalized pronominal forms may not be warranted (Johnston, 2013a,b).

However, one recent comparison of pronominal pointing in the BSL (British Sign Language) Corpus and the Tavis Smiley American English dataset found that the self- and other-directed pointing actions produced by deaf native signers of BSL are more conventionalized and reduced in form compared to those produced by hearing non-signing speakers of American English, although the function of these pointing acts requires further investigation (Fenlon et al., 2016; see also Cormier et al., 2013a; Goldin-Meadow and Brentari, 2015). Within a different language community, the Nheengatú of Brazil, Floyd (2016) found a quite conventional multimodal pattern used to reference time. In this community, speakers produce an auditory articulation coupled with a point to the sun’s position to refer to different times of day. Thus regardless of potential fine-grained differences across ecologies, it is evident that both signers and speakers systematically use bodily actions to index physical and abstract referents during their face-to-face interactions. These actions must therefore be included in a theory of language alongside forms that have received more attention from linguists, such as spoken or written deictic markers and pronominal forms, because they are all essential to understanding how humans signal through indicating.

Icons, in contrast to symbols and indices, partially depict meaning through perceptual resemblances (Clark, 1996). Signaling with icons is achieved through ‘demonstrations’ (Clark, 1996) or ‘depictions’ (Liddell, 2003). Paintings and drawings are prototypical examples of exhibited depictions, but here we focus on performed depictions co-created between signers and/or speakers (see Clark, 2016). More specifically, depictions are:

*[T]ypically iconic, representing what they stand for in terms of structural resemblances between form and meaning. They use material gradiently so that certain changes in form imply analogical differences in meaning. Consider the varying intensity of the strokes of paint that represent the shimmer and shadows on Magritte’s pipe, or the continuous movement of a hand gesture mimicking the trajectory of a ball. To interpret depictions, we imagine what it is like to see the thing depicted* (Dingemanse, 2015, p. 950).

Depiction signals icons that vary in their degree of conventionalization across a community. For instance, mimetic bodily enactments of people, animals or things (also known as ‘constructed action’ and ‘constructed dialog,’ Tannen, 1989; Metzger, 1995) used by signers and speakers to ‘show’ meaning rather than describe it (see Cormier et al., 2015b) are often analyzed as ‘singular events’ during which interactants interpret a form as ‘standing for’ a meaning within a specific usage event (Kockelman, 2005). These standing-for relations “become signs only when taken as signs in context” (Enfield, 2009, p. 13) (see the enactment by the Auslan signer in **Figure 1** as well as the constructed dialog produced by the English speaker in **Figure 5**).

Across the world’s signed languages, signs often called either ‘depicting’ signs (analyzed as partly lexical signs composed of conventional and non-conventional elements, see Liddell, 2003) or ‘classifier’ signs (analyzed as signed manifestations of the spoken or written classifier morphemes used in many spoken languages, see Supalla, 2003) represent another way signers can depict meanings. These signs have been a major focus of signed language research and describing and accounting for them within formal and structural theories of language presented an early challenge for signed language linguists (see e.g., Supalla, 1978; Klima and Bellugi, 1979), while others emphasized the iconic and context-dependent nature of these signs (e.g., DeMatteo, 1977; Johnston, 1989; Cogill-Koez, 2000). Researchers have observed that depicting signs are both iconically and spatially motivated while also exhibiting some level of conventionalization. They function to depict the handling of entities, the size and shape of entities, the location of entities, and the movement of entities (e.g., Liddell, 2003; Johnston and Schembri, 2007).

Depicting signs have been compared in varying degrees to the iconic and metaphoric manual gestures (also known as referential gestures) produced as part of spoken language discourse (see e.g., cf. Emmorey, 2003; Schembri et al., 2005; Streeck, 2009; Cormier et al., 2012). In addition, researchers investigating co-speech gesture have established fine-grained methods for detailing how hearing speakers depict with their hands and prompt meaning construction through different types of iconicity—often making a distinction between the hands as they depict the hands doing various activities vs. the hands depicting another type of referent (Müller, 1998, 2014, 2016; Kendon, 2004; Streeck, 2009). The types of gesture that result from these ‘modes of gestural representation’ are observed to align with the manual enactments and depicting signs observed across signed languages (Streeck, 2009; Müller, 2014).

The manual depictions briefly detailed above can be compared with ideophones. Dingemanse (2011, 2014, 2017a) explains that ideophones are spoken words that depict sensory imagery, and which are more or less integrated with surrounding morphosyntax. Examples include the Japanese *gorogoro* “rolling” and *kibikibi* “energetic” (mentioned in Dingemanse, 2017b). Ideophones function dually as descriptions and depictions, because of their conventionalized status, although novel ideophones can also be created within the context of an interaction. Others have compared ideophones to iconic, lexical signs in signed languages (e.g., Bergman and Dahl, 1994; Ferrara and Halvorsen, 2017). In “Composite Utterances Evidenced Within Hearing/Hearing interactions,” we will present an example from a Siwu language interaction that includes two examples of ideophones to illustrate the multimodal, composite utterances produced by hearing speakers.

Before discussing how *P*-signs are coordinated during face-to-face interaction, it is important to note that symbols, indices and icons are not exclusive categories—as illustrated by the introduction to ideophones above. Following Peirce, Clark (1996, p. 159) notes that “a single sign may have iconic, indexical, *and* symbolic properties” (emphasis in the original). For example, instances of enactment in which a speaker re-constructs an earlier dialog of themselves or another person might primarily be interpreted as depictions, but they are more precisely *depictions of prior acts of description*. Each depiction (via enactment) of the earlier event indexes both the original act of describing and any subsequent depiction of this event. Ideophones are fully conventional words that have both symbolic and iconic properties (Dingemanse, 2011). Signed language *P*-signs also exhibit multiple properties. Fully conventional lexical signs are descriptions, but in the case of more iconic lexical signs, they can also be used as depictions (e.g., the token of the lexical sign RUN in **Figure 2**, see also Johnston and Ferrara, 2012; Ferrara and Halvorsen, 2017). Other signs can be both symbolic and indexical, such as fully lexical signs that are meaningfully directed in space to index a referent (Liddell, 2003; Cormier et al., 2015a).

## Composite Utterances in Signed and Spoken Languages

Signers and speakers combine the three types of *P*-signs to ‘tell, show and do’ during face-to-face interactions. This occurs via the mutual orientation, recognition, and interpretation of social acts defined as communicative ‘moves.’ Within communicative moves, tokens of *P*-signs are temporally and spatially coordinated to create unified ‘composite utterances’ that are interpreted holistically rather than componentially (Enfield, 2009). A communicative move may be recognized as part of an interactional sequence, such as a turn, or more specifically as an instantiation of a type of linguistic utterance, such as an intonation unit or clause (see e.g., Thompson and Couper-Kuhlen, 2005). These moves are further defined by the temporal domain of ‘conversation time,’ i.e., the moment-by-moment temporality in which communicative moves unfold. Enfield (2009, p. 10) uses the term ‘enchrony’ to refer to conversation time and to differentiate it from historical time, i.e., diachrony.

As products of face-to-face interactions, composite utterances can be analyzed according to both their semiotic properties and the situated context of the interactions in which they emerge. With respect to their interpretation, it is the interaction of the elements within the composition that drives the creation – or rather, the disambiguation – of a “precise and vivid understanding” (Kendon, 2004, p. 174) more so than the use of language *per se* (see also Armstrong et al., 1995). The preciseness and vividness of an understanding, however, might be clarified by using more conventionalized semiotic resources such as lexicalized words or signs, to frame the less conventionalized properties of the utterance. For example, deaf signers’ strategic use of lexicalized signs to index and frame subsequent token enactments work to clarify who or what is being vividly enacted. In the same way, it is often the case that the visible bodily actions created by hearing speakers “cannot be precisely interpreted until [they are] perceived as part of the gesture-speech ensemble in which [they are] employed” (Kendon, 2004, p. 169). However, this relationship is reciprocal. For example, a hearing speaker’s enactment of throwing rice on the ground makes more salient the more vivid aspects of the verb ‘throw’ uttered in the speech, while the alignment of speech with the enacted actions simultaneously makes these actions more precise (see the relevant discussion of this example in Kendon, 2004, p. 169).

The literature on spoken languages, signed languages, semiotics, gesture studies, and anthropology attests to a wide range of evidence for the ubiquity of different *P*-signs and composite utterances across varied communicative ecologies. For example, the use of co-speech pointing actions to symbolically index physical and abstract referents – and very often their simultaneous temporal and semantic alignment with speech – have been described for diverse language ecologies such as the Cuna people of Panama (Sherzer, 1972), the Yupno people of Papua New Guinea and speakers of American English (Cooperrider et al., 2014), Murrinhpatha in Northern Australia (Blythe et al., 2016), Kreol Seselwa in the Seychelles (Brück, 2016), and speakers of Nheengatú in Brazil (Floyd, 2016). Across these ecologies, pointing is both a plurifunctional and multimodal referential strategy (integrating bodily actions, posture orientations and eye gaze either with or without speech) that patterns along formal, semantic, and spatiotemporal lines.

Additional research into hearing speaker’s use of co-speech gesture has shown that speakers’ manual gestures offer either complementary or supplementary semantic information, or perform the same pragmatic function, as the spoken utterance (McNeill, 1992; Goldin-Meadow, 2003; Kendon, 2004; Calbris, 2011). Other manual gestures often co-occur with speech in various ways to achieve nuanced semantic understandings. Kita and Özyürek’s (2003) cross-linguistic comparison of speech and gesture ensembles produced during elicited narratives in Turkish, English, and Japanese found that speakers of all three languages consistently produce manual depictions of the same motion events. The exact manifestation of depicting actions varies between languages and appears to be shaped by grammatical structure (i.e., linguistic packaging), the lexical content of the speech utterance, and also spatial information in the elicited materials that was never expressed verbally in the speech acts. Loehr’s (2012) analysis of the integration of intonation and manual gestures produced by English speakers indicates there is a strong temporal, structural, and pragmatic synchrony between speaker’s speech and gestural production. For example, Loehr describes how one hearing English speaker uses manual gesture and a steep L + H^∗^ pitch accent to highlight a contrast between a present state being described and an earlier one (Loehr, 2012, pp. 84–85).

It has also long been observed that tokens of manual depictions or bodily enactments may replace constituent ‘slots’ in spoken composite utterances that are usually ‘occupied’ by conventionalized words (Slama-Cazacu, 1973; Kendon, 1988; McNeill, 2012). Slama-Cazacu (1973, p. 180) described this process as producing a “mixed syntax” within the interaction. Ladewig’s (2014) research on manual gestures that replace speech within an utterance demonstrate how such gestures may function as verbs and nouns and are understood partly through the surrounding speech. She uses these observations as further evidence that language is multimodal. Clark (2016) explains that depictions are a part of everyday utterances and that they may function as various types of constituents (e.g., a noun phrase, an object of a verb, a non-restricted relative clauses) or independently. The use of enactment in spoken language interactions has also been shown to co-occur and interact with the more conventional aspects of speech (Sidnell, 2006; Keevallik, 2018) – particularly when it is used for direct quotation (Bolden, 2004; Park, 2009; De Brabanter, 2010; Sams, 2010). Comparable patterns have also been described for signed language interactions (e.g., Metzger, 1995; Quinto-Pozos, 2007; Cormier et al., 2013b; Ferrara and Johnston, 2014).

Although not undertaken explicitly using a composite utterance approach, one investigation of clause structure in FinSL (Finnish Sign Language) found that deaf signers use variable constituent order and frequently omit overt argument expression from their utterances (Jantunen, 2008). Jantunen (2008, p. 112) also identified ample evidence of “important pantomimic aspects,” i.e., enactment, which could not be handled in existing frameworks for analyzing clause structure. Indeed, corpus-based analysis of the clause-like composite utterances in elicited retellings by deaf signers of Auslan has shown that tokens of enactment are frequently and tightly integrated into Auslan syntax at the clause level, e.g., a token of enactment may function as a core predicate constituent. Signers also use enactment to elaborate aspects of their narratives that are encoded lexically and may even rely solely on enactment to show and infer semantic relations between participants and events in a story, instead of explicitly encoding these relations via fully lexicalized manual signs and other conventionalized strategies of morphosyntax (Ferrara and Johnston, 2014; Hodge and Johnston, 2014). In some ways, these findings mirror findings on the integration of enactment and gesture in spoken language discourse mentioned above.

Investigations of BSL and Auslan have found that signers typically frame their enactments with lexical noun phrases and/or pointing actions, which function to index the referent subsequently enacted with the signer’s body (Cormier et al., 2013b; Ferrara and Johnston, 2014). Ferrara (2012) analyzed more than 5,000 composite utterances containing depicting signs produced by Auslan signers during elicited retellings and conversational activities. She found that these tokens of partly lexical signs often combined with other types of signs, but could also stand alone as full utterances. Another corpus-based analysis of approximately 1,000 clause-like composite utterances produced by Auslan signers during elicited retellings found that one in three tokens of core argument or predicate expression in single, stand-alone utterances was a partly-lexical pointing or depicting sign, or a token of enactment (Hodge and Johnston, 2014). More recently, Janzen (2017) has discussed topic-comment constructions and perspective-taking constructions (i.e., character viewpoints versus signer-as-narrator viewpoints) in American Sign Language (ASL) as composite utterances.

These studies illustrate how some patterns of argument structure and multimodal utterance composition constitute strategies of situated co-construction that emerge as the interactions unfold, and are therefore highly dependent on the spatiotemporal context for recognition and interpretation. Given the essential role that indicating and depicting plays in signed interactions, these methods of signaling must be accounted for in signed language theory – as indeed they have been, albeit in various ways. We have seen that speakers also engage these methods of signaling. Thus, as signers and speakers both integrate multimodal indications and depictions into their utterances alongside descriptions in fairly conventional ways, a robust theory of language must be able to account for all three methods of signaling, even though token forms may vary in degree of conventionalization and how they are expressed across various language ecologies.

In the following sections, examples of composite utterances from deaf and hearing interactions are presented and discussed. First, two brief examples from interactions between deaf people are presented to illustrate how signers coordinate different types of *P*-signs within signed composite utterances. We then present an extended example that shows how deaf signers describe, indicate, and depict across longer stretches of interaction. In later sections, these examples are compared with examples from interactions between hearing speakers. Our aim is to demonstrate the coordinated signaling of description, depiction, and indication evidenced in both signed and spoken language interactions and achieve comparable analyses for both. We argue that Clark’s theory of language use is a strong starting point for uniting the communicative practices emerging within diverse ecologies under one theory of language. In this way, we extend Clark’s theory of language use to a theory of language.

## Composite Utterances Evidenced Within Deaf/Deaf Interactions

A first example of a composite utterance evidenced in a deaf/deaf interaction is produced by a deaf Auslan signer re-telling *Frog, Where Are You?* (Mayer, 1969) to another deaf signer (**Figure 1**). During the story, a little boy searches for his missing pet frog. In retelling one moment of the story, the signer produces a composite utterance that both depicts and describes the boy as he picks up a boot and looks inside it. The signer begins with an enactment of the boy holding something over his head (i.e., a depiction), using eye gaze and facial orientation to index an as-yet un-named referent to a specific location in the signing space. This enactment is followed by a fingerspelled English word (‘boot’) and the lexical Auslan sign BOOT (i.e., a description of the object held by the boy). The signer completes his move with another enactment of the boy holding up the boot and looking into it (again, simultaneously depicting the event and indexing referents within the event). In this way, the signer coordinates different acts of description, indication, and depiction to create a composite utterance recounting a moment in the boy’s search for the frog. The initial enactment is elaborated retrospectively through the description of the referent ‘boot’ in both English and Auslan. The second iteration of the enactment enables the signer’s interactant to once again perceive what happened, but with clarified knowledge about the imagined object the boy (or rather, the signer as boy) was holding. In this composite utterance, the descriptions, indications, and depictions are essential to understanding the meaning. Without the depictions, for example, all that would remain is a (bilingual) description of the referent ‘boot,’ which does little to move the story forward. In this example, we see that the availability of bodily enactment precludes the need to formulate a description through fully conventionalized lexis and grammar. We contend that such practices, based in the essentially face-to-face nature of interaction, have been able to fundamentally shape the signed languages of deaf communities (Johnston, 1996).

A second example further illustrates the nature of signed language communication by detailing a composite utterance produced as part of an informal conversation between three deaf Norwegians (**Figure 2**). The signer has almost finished detailing a personal experience about her childhood. She recounts how her father would have to physically come and find her when she was out playing, because she could not hear his calls. Her utterance begins with the signs POINT FATHER, thereby naming ‘father’ as the actor referent. The pointing action serves to index her own father, as opposed to someone else’s. The signer then elaborates her father’s actions by exploiting the gradient properties of the fully conventionalized sign RUN to express how her father would have to run (and find her). Her skillful manipulation of this lexical sign has the effect of profiling both descriptive and depictive elements of her expression. She ends this composite utterance by enacting her father as he ran to her, reached out and physically took hold of her, thus also indexing her young self as a referent through eye gaze and meaningful use of space. This depiction (which essentially functions as a verb) is framed by the phrase that both indicates and describes her father as the actor referent. As in the Auslan example, these descriptions, indications, and depictions are all integral to the intended meaning and must be interpreted holistically. If we were to focus only on the most conventionalized aspects of this utterance, i.e., the descriptive signs FATHER and RUN, then we would be left with only a partial understanding and analysis.

**FIGURE 3:**
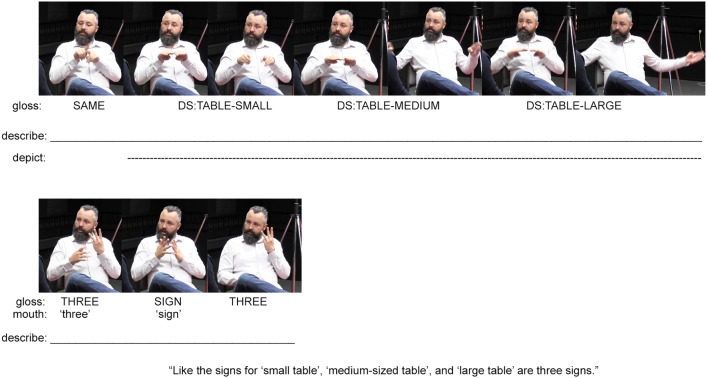
An example of an Auslan signer indicating, describing, and depicting across composite utterances (images used with consent, see Hodge et al., forthcoming for information about this dataset).

**FIGURE 4:**
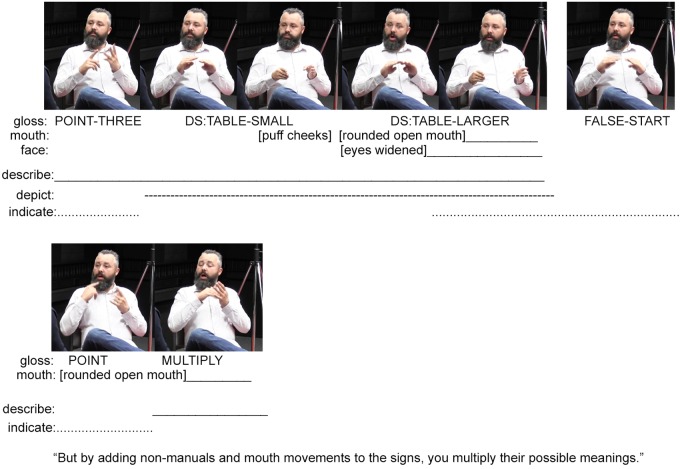
A continuation of the Auslan example in **Figure 3** (images used with consent).

These two brief examples illustrate how deaf signers produce descriptions, indications, and depictions through manual and non-manual actions within composite utterances to express complex meanings. These methods of signaling cannot be easily isolated or divided from each other: they must be accounted for as integrated signals. The processes of describing, indicating, and depicting can be further clarified by examining an extended interaction between two deaf signers conversing in Auslan, i.e., a sequence of communicative moves comprising an interactional event (**Figures 3**, **4**). Both signers are teachers of Auslan in Melbourne, engaging in a metalinguistic discussion about the strategies signers use to exploit and expand the comparably small lexicons of signed languages. This example consists of five composite utterances over 8 s. It was documented during the conversation task session for the Auslan and Australian English Corpus (Hodge et al., forthcoming).

The signer begins by producing three modified iterations of the fully conventionalized sign TABLE. By manipulating the depictive characteristics of the symbol TABLE, i.e., the resemblance in shape to a prototypical table, each iteration differentiates three tables of different sizes (**Figure 3**). Signers exploit the iconic nature of signs in such ways as to manipulate meaning construction, and in doing so, they profile the dual function of many signs as descriptions and depictions (see also the sign RUN in the Norwegian Sign Language example in **Figure 2**; Johnston and Ferrara, 2012; Ferrara and Halvorsen, 2017). Comparable manipulations of iconic words have been observed in spoken languages (e.g., Dingemanse and Akita, 2016; Dingemanse, 2017a), which points to interesting similarities and differences between signed and spoken language ecologies.

Although these are just three versions of the lexical sign TABLE, the signer further explains that with different non-manual actions, one can multiply the meanings of these three signs. He does this by first describing his previous actions as ‘three signs’ via the fully conventional lexical signs (THREE SIGN THREE) and mouthings of the conventional English words ‘three’ and ‘sign’ (also illustrated in **Figure 3**). Using his right hand, he then points to the sign THREE, which was preserved on his left hand (**Figure 4**). This is possible because signers can hold signs over periods of time, creating possibilities of future interaction with those signs as physical entities. Although speakers are unable to hold spoken words over time while also continuing to speak, they can produce manual gestures that they interact with as physical entities.^2^ The signer’s point to the sign THREE indexes the three signs depicting the differently-sized tables produced earlier. He then repeats these depictions while adding various mouth movements and non-manual actions to this reproduction (see the top row in **Figure 4**). The signer concludes by explaining that these non-manual actions “multiply the meanings of signs” (thus justifying why deaf signed languages do not require extensive manual signed lexicons). This explanation is expressed through a pointing sign that indicates his mouth (and thereby indexes the various movements undertaken during the preceding depictions) and a description (the lexical sign MULTIPLY), which explains the multiplying effect such non-manuals have on the meanings of signs. Again, this example demonstrates how methods for description, indication, and depiction are integrated within composite utterances. By focusing on one method of signaling only, we are unable to account for the full expression of the utterance – too much would be left out.

The three examples presented in this section show that deaf signers make strategic choices during the co-creation of composite utterances. Face-to-face interaction allows for the extensive use of all three methods of signaling, but particularly promotes the use of methods for indicating and depicting. The availability of space in deaf signed language interactions, we have seen, means that signers often rely heavily on indication and depiction for meaning construction. This has implications for the use of descriptions as well as the development of the inventory of conventionalized symbols which emerge within ecologies that are primarily (or in the case of deaf signed interactions, exclusively) face-to-face. Thus, theories of language which account only for conventionalized symbolic forms and the descriptions that signal them are incomplete, while also hindering an accurate understanding of how description works in combination with the other two methods of signaling (see also Liddell, 2003, p. 362). Furthermore, the research reviewed in earlier sections has illustrated how hearing speakers also engage all three methods of signaling. One possible way to unite this knowledge into a global theory of language is to extend Clark’s theory of (spoken) language use to that of language more generally, thus integrating findings from signed language linguistics, gesture research, and other disciplines into linguistic theory. More importantly, we can begin to understand how diverse humans communicate with each other without drawing haphazard and somewhat arbitrary lines around what is ‘linguistic’ and ‘non-linguistic.’ In the next section, we examine some examples of composite utterances evidenced in spoken language interactions to further demonstrate this position.

## Composite Utterances Evidenced Within Hearing/Hearing Interactions

In this section, we turn our focus to examples of composite utterances produced during interactions between hearing speakers. By contrasting the composite utterances produced during deaf/deaf interactions with those produced during hearing/hearing interactions, we can begin to consider exactly how the communicative ecologies of signers and speakers may shape their coordination of methods for describing, indicating, and depicting within composite utterances. Firstly, an example from the literature briefly illustrates how hearing speakers create semantic and structural synchrony within their multimodal composite utterances:

[1] Ideophones and co-occurring manual gesture integrated with Siwu speech utterances (Dingemanse, 2014, p. 392):

gɔ ɔ-nyà ɔ-s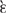 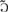-ã′-bo,

when 3sg-see 3sg-hab 3sg-fut-reach

gɔ ɔ-nyà ɔdi àra,

when 3sg-see 3sg-take things,

“*So when he got there, when he undressed*,

gɔ ɔ-nyà kùgɔ ɔ-nya, ↑↑walayayayayaya↑↑

when 3sg-see how 3sg-see, idph.walayayayayaya

*just when he’s about to - walayayayayaya*!” ((gestures waves of water passing over skin))

oh, ɔ-tsùè pelepelepelepele

oh, 3sg:pst-burn idph.completely

“*Oh, he was scalded all over*.”

In Example [1], the Siwu speaker depicts what happened to the king during an unfortunate bath by using conventional and non-conventional ideophones (*walayayayayaya* and *pelepelepelepele*) and manual gesture, while also describing what happened using fully conventionalized Siwu words and grammatical constructions. There are also examples of deictic morphemes (ɔ) that indicate the king as referent.

Similarly, Green and Wilkins (2014, p. 252) investigated the alternate signed language practices used by Arandic speaking communities of Central Australia and found that speakers habitually coordinate composite, multimodal packages with and without speech. These composite utterances involve different semiotic elements, including graphic depictions drawn in the sand, spoken words, and conventionalized signs produced with the hands, whereby each element serves to disambiguate the others. These patterns are akin to the ways in which Australian and Norwegian deaf signers use fully conventionalized signs and words to disambiguate their bodily enactments (see Composite Utterances Evidenced Within Deaf/Deaf Interactions). In each case, both signers and speakers make strategic, moment-by-moment choices about how to disambiguate the context of the interaction and prompt meaning construction, and then execute these choices by drawing from their available semiotic repertoire. A theory of language should be fully compatible with these choices by including both emerging and established communicative practices.

**FIGURE 5:**
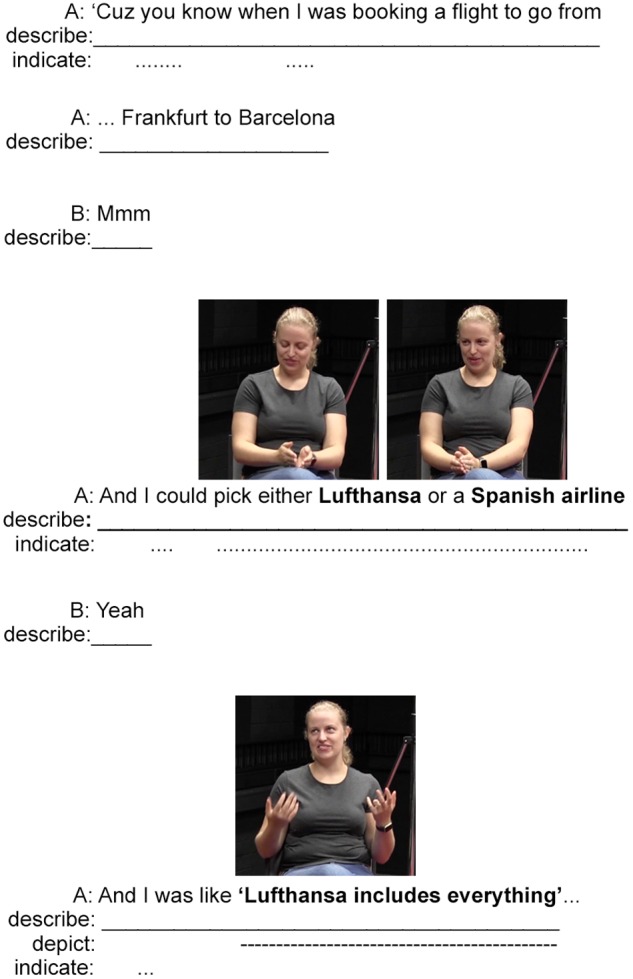
An example of an Australian English speaker describing, indicating, and depicting across composite utterances (images used with consent, see Hodge et al., forthcoming for information about this dataset).

**FIGURE 6:**
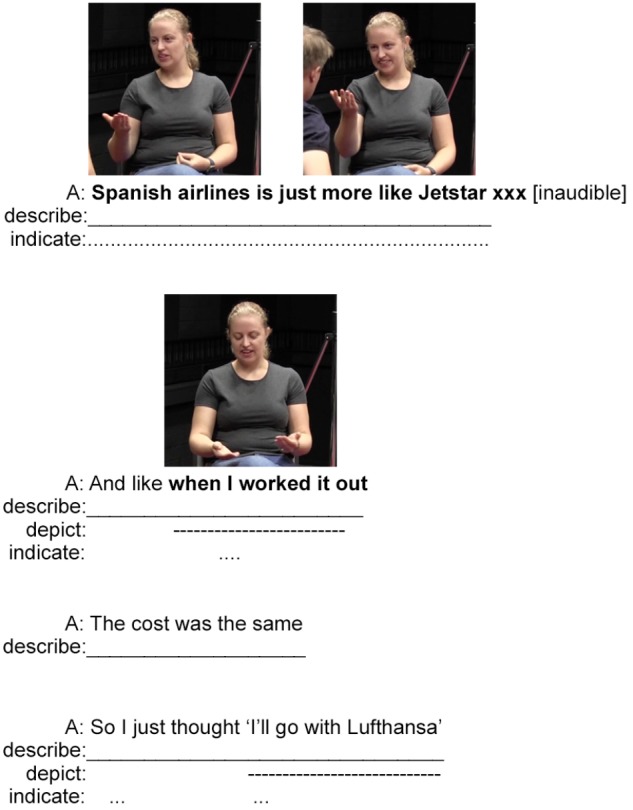
A continuation of the Australian English example in **Figure 5** (images used with consent).

The next example involves composite utterances produced during an informal conversation between two hearing Australian English speakers. It was documented during the conversation task session for the Auslan and Australian English Corpus (Hodge et al., forthcoming). During this interaction, a hearing woman is chatting to her brother about a previous experience booking a flight for travel in Europe. She explains how she compared two airlines and discovered that the low-cost airline was not so low-cost after all. She does this by coordinating her speech, hand, and body in acts of description, indication, and depiction. This example is presented in **Figures 5**, **6** with relevant images of meaningful hand and body movements aligned with co-occurring periods of speech (represented in bold).

The example begins in **Figure 5** with two utterances that introduce the topic through a description using spoken English lexis and syntax: “*Cuz you know when I was booking a flight to go from …Frankfurt to Barcelona*.” The speaker then makes eye-contact with her brother (who was engaged with picking up a glass and taking a sip of water while she spoke) as he provides a confirmatory “*Mmm*.” She continues with a composite utterance that describes with speech the possible choice of two airlines. As she names the two airlines, she produces hand movements to indicate the two choices and locate the choices in space. These pointing actions also work to set the two choices up in opposition: she points her hands joined at the fingertips to the left of her leg to indicate Lufthansa, and then to the right to indicate a Spanish airline. This multimodal, composite utterance effectively presents the topic of conversation—namely a comparison of two airlines—through acts of description and indication.

In the next composite utterance, the speaker presents the first part of her comparison by combining speech, hand and head movements, and facial expression to describe and depict her thought process (*And I was like* “*Lufthansa includes everything*”). The utterance begins with the English construction *And I was like*, which works to frame the subsequent depiction of (presumably) a thought process. The spoken part of this depiction is synchronized with the speaker raising her hands and shifting her gaze upward to demonstrate that the price from Lufthansa would be all-inclusive. Her hand movements in this utterance resemble what Kendon (2004) refers to as the Open Hand Supine gesture (OHS, in this case, a two-handed version), which has been analyzed as a gesture that relates to acts of receiving. Here, we can interpret this gesture as contributing to the meaning of the depiction that one receives everything included with a Lufthansa ticket, which may justify its higher initial price.

The speaker’s next composite utterance works to link the current interaction back to earlier comments her brother had made about the Australian low-cost airline Jetstar. She begins with a very brief manual indication to the Spanish airlines by producing another instance of an OHS gesture (this time on only the right hand) that she places on her right – notably, in the same space that indicated the Spanish airlines at the beginning of the example. Without directed movement, we may interpret this gesture as a Palm Presenting version of the OHS that presents the Spanish airlines as a focus. However, its function to indicate the Spanish airlines through meaningful location in space may mean this gesture is best analyzed as a Palm Addressed OHS gesture (see Kendon, 2004, Chapter 13). In any case, the gesture is accompanied by, and elaborated upon, with a description in spoken English, “*Spanish airlines*.” This phrase is followed by further description in spoken English that clarifies that the Spanish airline is similar to Jetstar. As the speaker utters this description, she once again produces an OHS gesture; this time a clearer example of the Palm Addressed type. She moves this gesture toward her brother, while also shaking the hand laterally, effectively acknowledging and indicating his earlier comments about Jetstar and their hidden costs.

The speaker then continues with two composite utterances that describe with speech the calculations she did to compare the costs between the airlines: “*And like when I worked it out, the cost was the same.*” While uttering these descriptions, the speaker also synchronizes a co-speech manual depiction comparing the two prices. This manual gesture can possibly be analyzed as depicting the ‘weighing of objects’ on a scale—the hands representing the surfaces of the two sides of the scale, which objects are placed upon [i.e., Müller’s (2014) representing gestures or Kendon’s (2004) modeling gestures]. An alternative analysis interprets the two hands as two calculations, again, representing gestures, that allow the speaker to visually inspect the choices. This example concludes with a framed depiction of the speaker’s final decision: *So I just thought* “*I’ll go with Lufthansa.*”

Overall, the acts of description, indication, and depiction coordinated within these composite utterances are very similar to the signaling acts produced during deaf/deaf interactions detailed in Section “Composite Utterances Evidenced Within Deaf/Deaf Interactions” and the hearing speaker in example [1] above. However, one difference between deaf/deaf and hearing/hearing interactions is immediately apparent: speakers recruit speech and sound into their composite utterances in addition to visible bodily actions, whereas deaf signers typically only do this when they know the other person can hear. This fundamental difference reflects the respective lifeworlds and communicative ecologies of deaf and hearing people. The availability sound, or lack thereof, has important implications for analyzing and comparing signed and spoken interactions.

## Re-Orienting Language Theory to Reflect Multimodal Language as Action

In this paper, we have extended Clark’s (1996) theory of language use to acknowledge that language and language use cannot be divided and to account for the diverse yet comparable communication practices which emerge during deaf/deaf and hearing/hearing interactions. As Dingemanse (2017b, p. 195) has commented, the tools we use to investigate language (i.e., our methods and theories) “enhance our powers of observation at one level of granularity (at the expense of others), and they bring certain phenomena in focus (defocusing others).” He suggests that sometimes these tools need to be recalibrated. In this paper, we have proposed re-calibrating traditional, structural theories of language with a more holistic theory that conceptualizes language as ‘actioned’ via three methods of signaling: describing, indicating and depicting. Evidence from the existing literature on signed and spoken languages demonstrates that these three methods of signaling are essential to understanding face-to-face communication. We have shown how both deaf signers and hearing speakers describe, indicate, and depict within composite utterances. In addition to signaling through description, both signers and speakers signal through indication and depiction within the spatiotemporal context of their unfolding interactions, although the exact manifestations of these patterns diverge according to the availability of sound. These patterns attest to the pluralistic complexity of human communication and the varied semiotic repertoires which emerge within specific language ecologies. If we are to strive for robust and complex understandings of both signed and spoken language use, any language theory must acknowledge the broad range of intentionally expressive actions available to agents within specific spatiotemporal contexts, and the complex ecologies in which signers and speakers live. This can be achieved through direct comparison of the ways in which diverse humans produce and coordinate acts of description, indication, and depiction during their face-to-face interactions.

## Author Contributions

LF conceptualized the study, gathered the data, analyzed the data, and wrote 50% of the manuscript. GH detailed the theoretical argumentation, checked analysis of data, and wrote 50% of the manuscript.

## Conflict of Interest Statement

The authors declare that the research was conducted in the absence of any commercial or financial relationships that could be construed as a potential conflict of interest.

## Footnotes

1 Clark (1996) first proposed ‘describing-as,’ ‘indicating’ and ‘demonstration’ as the names of the three methods of signaling. Here we abbreviate ‘describing-as’ to ‘describing’ and use the term ‘depiction’ instead of ‘demonstration’ to correspond with more recent signed and spoken language literature (Liddell, 2003; Dudis, 2011; Dingemanse, 2014; Clark, 2016).

2 Our thanks to a reviewer for pointing this out.

## References

[B1] Agha A. (2005). Voice, footing, enregisterment. 15 38–59. 10.1525/jlin.2005.15.1.38

[B2] Agha A. (2007). Cambridge: Cambridge University Press.

[B3] Armstrong D. F. Stokoe W. Wilcox S. (1995). Cambridge: Cambridge University Press 10.1017/CBO9780511620911

[B4] Bavelas J. B. Hutchinson S. Kenwood C. Matheson D. H. (1997). Using face-to-face dialogue as a standard for other communication systems. 22 5–24. 10.22230/cjc.1997v22n1a973

[B5] Bergman B. Dahl Ö. (1994). “Ideophones in sign language? The place of reduplication in the tense-aspect system of Swedish Sign Language,” in , eds Bache C. Basbøll H. Lindberg C.-E. (Berlin: Walter de Gruyter), 397–422.

[B6] Bernstein B. (2003/1971). London: Routledge.

[B7] Blythe J. Mardigan K. Carmelita P. Mawurt E. Stoakes H. (2016). Pointing out directions in Murrinhpatha. 2 132–159. 10.1515/opli-2016-0007

[B8] Bolden G. (2004). The quote and beyond: defining boundaries of reported speech in conversational Russian. 36 1071–1118. 10.1016/j.pragma.2003.10.015

[B9] Bourdieu P. (1991). Cambridge, MA: Harvard University Press.

[B10] Bressem J. (2013). “20th century: empirical research of body, language, and communication,” in , eds Müller C. Cienki A. Fricke E. Ladewig S. H. McNeill D. Tessendorf S. (Berlin: Mouton De Gruyter), 393–416.

[B11] Brück M. A. (2016). Ph.D. dissertation, Universität zu Köln, Köln Available at: http://kups.ub.uni-koeln.de/id/eprint/7964

[B12] Busch B. (2015). Expanding the notion of the linguistic repertoire: on the concept of spracherleben—the lived experience of language. 38 340–358. 10.1093/applin/amv030

[B13] Bühler K. (1990/1934). Philadelphia, PA: John Benjamins.

[B14] Calbris G. (2011). *Elements of Meaning in Gesture*, trans. Copple M. M. Amsterdam: John Benjamins 10.1075/gs.5

[B15] Cienki A. Müller C. (eds) (2008). Amsterdam: John Benjamins 10.1075/gs.3

[B16] Clark H. H. (1996). Cambridge: Cambridge University Press 10.1017/CBO9780511620539

[B17] Clark H. H. (2003). “Pointing and placing,” in , ed. Kita S. (Mahwah, NJ: Lawrence Erlbaum Associates), 243–268.

[B18] Clark H. H. (2016). Depicting as a method of communication. 123 324–347. 10.1037/rev0000026 26855255

[B19] Cogill-Koez D. (2000). A model of signed language ‘classifier predicates’ as templated visual representation. 3 209–236. 10.1075/sll.3.2.04cog

[B20] Cooperrider K. Núñez R. Slotta J. (2014). “The protean pointing gesture: variation in a building block of human communication,” in , eds Bello P. Guarini M. McShane M. Scassellati B. (Austin, TX: Cognitive Science Society), 355–360.

[B21] Cormier K. Fenlon J. Schembri A. (2015a). Indicating verbs in British Sign Language favour use of motivated space. 1 684–707. 10.1515/opli-2015-0025

[B22] Cormier K. Quinto-Pozos D. Sevcikova Z. Schembri A. (2012). Lexicalisation and de-lexicalisation processes in sign languages: comparing depicting constructions and viewpoint gestures. 32 329–348. 10.1016/j.langcom.2012.09.004 23805017 PMC3688355

[B23] Cormier K. Schembri A. Woll B. (2013a). Pronouns and pointing in sign languages. 137 230–247. 10.1016/j.lingua.2013.09.010

[B24] Cormier K. Smith S. Sevcikova Z. (2015b). Rethinking constructed action. 18 167–204. 10.1075/sll.18.2.01cor

[B25] Cormier K. Smith S. Zwets M. (2013b). Framing constructed action in British Sign Language narratives. 55 119–139. 10.1016/j.pragma.2013.06.002

[B26] De Brabanter P. (2010). The semantics and pragmatics of hybrid quotations. 4 107–120. 10.1111/j.1749-818X.2009.00185.x

[B27] DeMatteo A. (1977). “Visual imagery and visual analogues in American Sign Language,” in , ed. Friedman L. A. (New York, NY: Academic Press), 109–136.

[B28] Dingemanse M. (2011). Ideophones and the aesthetics of everyday language in a west-African society. 6 77–85. 10.2752/174589311X12893982233830

[B29] Dingemanse M. (2014). Making new ideophones in Siwu: creative depiction in conversation. 5 384–405. 10.1075/ps.5.3.04din

[B30] Dingemanse M. (2015). Ideophones and reduplication: depiction, description, and the interpretation of repeated talk in discourse. 39 946–970. 10.1075/sl.39.4.05din

[B31] Dingemanse M. (2017a). Expressiveness and system integration: on the typology of ideophones, with special reference to Siwu. 70 119–141. 10.1515/stuf-2017-0018

[B32] Dingemanse M. (2017b). “On the margins of language: ideophones, interjections and dependencies in linguistic theory,” in , ed. Enfield N. J. (Berlin: Language Science Press), 195–203.

[B33] Dingemanse M. Akita K. (2016). An inverse relation between expressiveness and grammatical integration: on the morphosyntactic typology of ideophones, with special reference to Japanese. 53 501–532. 10.1017/S002222671600030X

[B34] Dudis P. (2011). “The body in scene depictions,” in , ed. Roy C. (Washington, DC: Gallaudet University Press), 3–45.

[B35] Duranti A. Goodwin C. (eds) (1992). Cambridge: Cambridge University Press.

[B36] Emmorey K. (ed.) (2003). Mahwah, NJ: Lawrence Erlbaum Associates.

[B37] Enfield N. J. (2009). Cambridge: Cambridge University Press 10.1017/CBO9780511576737

[B38] Enfield N. J. (2013). Oxford: Oxford University Press 10.1093/acprof:oso/9780199338733.001.0001

[B39] Everett D. L. (2005). Cultural constraints on grammar and cognition in Pirahã: another look at the design features of human language. 46 621–646. 10.1086/431525 21205333

[B40] Fenlon J. Keane J. Cooperrider K. J. Brentari D. Goldin-Meadow S. (2016). Comparing pronominal signs with pointing gestures. , Paris.

[B41] Fenlon J. Schembri A. Kearsy C. (2018). Modification of indicating verbs in British Sign Language: a corpus-based study. 94 84–118. 10.1353/lan.2018.0002

[B42] Ferrara L. (2012). Ph.D. dissertation, Macquarie University, Sydney, NSW.

[B43] Ferrara L. Bø V. (2015). Trondheim: NTNU & HiOA.

[B44] Ferrara L. Halvorsen R. P. (2017). Depicting and describing meanings with iconic signs in Norwegian Sign Language. 16 371–395. 10.1075/gest.00001.fer

[B45] Ferrara L. Johnston T. (2014). Elaborating who’s what: a study of constructed action and clause structure in Auslan (Australian Sign Language). 34 193–215. 10.1080/07268602.2014.887405

[B46] Floyd S. I. (2016). Modally hybrid grammar? Celestial pointing for time-of-day reference in Nheengatú. 92 31–64. 10.1353/lan.2016.0013

[B47] Fricke E. (2014). “Deixis, gesture, and embodiment from a linguistic point of view,” in , eds Müller C. Cienki A. Fricke E. Ladewig S. H. McNeill D. Bressem J. (Berlin: Mouton De Gruyter), 1802–1823.

[B48] Goldin-Meadow S. (2003). Cambridge, MA: Harvard University Press.

[B49] Goldin-Meadow S. Brentari D. (2015). Gesture, sign and language: the coming of age of sign language and gesture studies. 40:e46. 10.1017/S0140525X15001247 26434499 PMC4821822

[B50] Goodwin C. (2000). Action and embodiment within situated human interaction. 32 1489–1522. 10.1016/S0378-2166(99)00096-X

[B51] Green E. M. (2015). “‘One language, or maybe two: direct communication, understanding, and informal interpreting in international deaf encounters’,” in , eds Friedner M. Kusters A. (Washington, DC: Gallaudet University Press), 70–82.

[B52] Green J. A. (2014). Cambridge: Cambridge University Press 10.1017/CBO9781139237109

[B53] Green J. A. Wilkins D. P. (2014). With or without speech: Arandic sign language from Central Australia. 34 234–261. 10.1080/07268602.2014.887407 18816421

[B54] Harrelson E. M. (2017). Deaf people with “no language”: mobility and flexible accumulation in languaging practices of deaf people in Cambodia. 10.1515/applirev-2017-0081 [Epub ahead of print].

[B55] Haugen E. (1972). Stanford, CA: Stanford University Press.

[B56] Hodge G. (forthcoming). “The ideology of communication practices embedded in an Australian deaf/hearing dance collaboration,” in , eds Kusters A. Green M. Harrelson E. M. Snoddon K. (Berlin: Mouton de Gruyter).

[B57] Hodge G. Johnston T. (2014). Points, depictions, gestures and enactment: partly lexical and non-Lexical signs as core elements of single clause-like units in Auslan (Australian Sign Language). 34 262–291. 10.1080/07268602.2014.887408

[B58] Hodge G. Sekine K. Schembri A. Johnston T. (forthcoming). Comparing signers and speakers: building a directly comparable corpus of Auslan and Australian English. 14.

[B59] Jantunen T. (2008). Fixed and free: order of the verbal predicate and its core arguments in declarative transitive clauses in Finnish Sign Language. 21 83–123.

[B60] Janzen T. (2017). Composite utterances in a signed language: topic constructions and perspective-taking in ASL. 28 511–538. 10.1515/cog-2016-0121

[B61] Johnston T. (1996). “Function and medium in the forms of linguistic expression found in a sign language,” in Vol. 1 eds Edmondson W. H. Wilbur R. B. (Mahwah, NJ: Lawrence Erlbaum), 57–94.

[B62] Johnston T. (2008). “The Auslan archive and corpus,” in , ed. Nathan D. (London: University of London).

[B63] Johnston T. (2012). Lexical frequency in sign languages. 17 163–193. 10.1093/deafed/enr036 21841168

[B64] Johnston T. (2013a). Formational and functional characteristics of pointing signs in a corpus of Auslan (Australian sign language): are the data sufficient to posit a grammatical class of ‘pronouns’ in Auslan? 9 109–159.

[B65] Johnston T. (2013b). Towards a comparative semiotics of pointing actions in signed and spoken languages. 13 109–142. 10.1075/gest.13.2.01joh

[B66] Johnston T. (ed.) (1989). Sydney, NSW: Deafness Resources Australia.

[B67] Johnston T. Ferrara L. (2012). “Lexicalization in signed languages: when an idiom is not an idiom,” in *Proceedings the 3rd UK Cognitive Linguistics Conference*, Vol. 1 Hatfield, 229–248.

[B68] Johnston T. Schembri A. (2007). Cambridge: Cambridge University Press.

[B69] Keevallik L. (2018). What does embodied interaction tell us about grammar? 51 1–21. 10.1080/08351813.2018.1413887

[B70] Kelly B. F. (2002). “The development of speech, gesture, and action as communicative strategies,” in (Berkeley, CA: University of California).

[B71] Kelly B. F. (2006). “The development of constructions through gesture,” in , eds Clark E. V. Kelly B. F. (Palo Alto, CA: CSLI), 11–25.

[B72] Kendon A. (1988). “How gestures can become like words,” in , ed. Poyatos F. (Toronto, ON: C.J. Hogrefe Publishers), 131–141.

[B73] Kendon A. (2004). Cambridge: Cambridge University Press.

[B74] Kendon A. (2008). Some reflections on the relationship between ‘gesture’ and ‘sign’. 8 348–366. 10.1075/gest.8.3.05ken

[B75] Kendon A. (2014). Semiotic diversity in utterance production and the concept of ‘language’. 369:20130293. 10.1098/rstb.2013.0293 25092661 PMC4123672

[B76] Kita S. (2003). “Pointing: a foundational building block of human communication,” in , ed. Kita S. (Mahwah, NJ: Lawrence Erlbaum Associates), 1–8.

[B77] Kita S. Özyürek A. (2003). What does cross-linguistic variation in semantic coordination of speech and gesture reveal?: evidence for an interface representation of spatial thinking and speaking. 48 16–32. 10.1016/S0749-596X(02)00505-3

[B78] Klima E. Bellugi U. (1979). Cambridge, MA: Harvard University Press.

[B79] Kockelman P. (2005). The semiotic stance. 157 233–304. 10.1515/semi.2005.2005.157.1-4.233

[B80] Kral I. Ellis E. M. (2008). “‘Children, language and literacy in the Ngaanyatjarra lands’,” in , eds Simpson J. Wiggelsworth G. (London: Continuum), 154–172.

[B81] Kravchenko A. (2006). Cognitive linguistics, biology of cognition and biosemiotics: bridging the gaps. 28 51–75. 10.1016/j.langsci.2005.02.002

[B82] Kusters A. (2017). “Our hands must be connected”: visible gestures, tactile gestures and objects in interactions featuring a deafblind customer in Mumbai. 27 394–410. 10.1080/10350330.2017.1334386

[B83] Kusters A. Spotti M. Swanwick R. Tapio E. (2017). Beyond languages, beyond modalities: transforming the study of semiotic repertoires. 14 219–232. 10.1080/14790718.2017.1321651

[B84] Ladewig S. H. (2014). “Creating multimodal utterances: the linear integration of gestures into speech,” in , eds Müller C. Cienki A. Fricke E. Ladewig S. H. McNeill D. Bressem J. (Berlin: De Gruyter Mouton), 1662–1677.

[B85] LaPolla R. (2003). “Why languages differ: variation in the conventionalization of constraints on inference,” in , eds Bradley D. LaPolla R. Michailovsky B. Thurgood G. (Canberra, ACT: Australian National University), 113–144.

[B86] LaPolla R. (2005). “Typology and complexity,” in , eds Minett J. W. Wang W.S.-Y. (Hong Kong: City University of Hong Kong Press), 465–493.

[B87] Levinson S. Enfield N. J. (2006). Oxford: Berg.

[B88] Liddell S. K. (1995). “Real, surrogate, and token space: grammatical consequences in ASL,” in , eds Emmorey K. Reilly J. (Hillsdale, NJ: Lawrence Erlbaum Associates), 19–41.

[B89] Liddell S. K. (2003). New York, NY: Cambridge University Press 10.1017/CBO9780511615054

[B90] Loehr D. P. (2012). Temporal, structural, and pragmatic synchrony between intonation and gesture. 3 71–89. 10.1515/lp-2012-0006

[B91] Maturana H. R. Varela F. J. (1987). Boston, MA: Shambhala Publications.

[B92] Mayer M. (1969). New York, NY: Dial Press.

[B93] McNeill D. (1992). Chicago, IL: University of Chicago Press.

[B94] McNeill D. (2012). Cambridge: Cambridge University Press 10.1017/CBO9781139108669

[B95] Metzger M. (1995). “‘Constructed dialogue and constructed action in American Sign Language’,” in , ed. Lucas C. (Washington, DC: Gallaudet University Press), 255–271.

[B96] Mittelberg I. (2008). “Peircean semiotics meets conceptual metaphor: Iconic modes in gestural representations of grammar,” in , eds Müller C. Cienki A. (Amsterdam: John Benjamins), 115–154.

[B97] Müller C. (1998). “Iconicity and gesture,” in , eds Cavé C. Guaitella I. Santi S. (Montréal/Paris: L’Harmattan), 321–328.

[B98] Müller C. (2014). “Gesture as deliberate expressive movement,” in , eds Seyfeddinipur M. Gullberg M. (Philadelphia: John Benjamins), 127–151.

[B99] Müller C. (2016). “From mimesis to meaning: a systematics of gestural mimesis for concrete and abstract referential gestures,” in , eds Zlatev J. Sonesson G. Konderak P. (Frankfurt: Peter Lang), 211–226.

[B100] Müller C. Cienki A. Fricke E. Ladewig S. H. McNeill D. Bressem J. (eds). (2014). Berlin: De Gruyter Mouton.

[B101] Müller C. Cienki A. Fricke E. Ladewig S. H. McNeill D. Tessendorf S. (eds). (2013). Berlin: De Gruyter Mouton.

[B102] Nevile M. Haddington P. Heinemann T. Rauniomaa M. (eds) (2014). Amsterdam: John Benjamins.

[B103] Okrent A. (2002). “A modality-free notion of gesture and how it can help us with the morpheme vs. gesture questions in sign language linguistics (Or at least give us some criteria to work with),” in , eds Meier R. Cormier K. Quinto-Pozos D. (New York, NY: Cambridge University Press), 175–198.

[B104] Park Y. (2009). Interaction between grammar and multimodal resources: quoting different characters in Korean multiparty conversation. 11 79–104. 10.1177/1461445608098499

[B105] Parmentier R. J. (1994). Indianapolis, IN: Indiana University Press.

[B106] Peirce C. S. (1955). New York, NY: Dover Publications.

[B107] Pennycook A. Otsuji E. (2014). Metrolingual multitasking and spatial repertoires: ‘Pizza mo two minutes coming’. 18 161–184. 10.1111/josl.12079

[B108] Perniss P. Özyürek A. Morgan G. (2015). The influence of the visual modality on language structure and conventionalization: insights from sign language and gesture. 7 2–11. 10.1111/tops.12127 25565249

[B109] Pickering J. (1997). “Beyond cognitivism: mutualism and postmodern psychology,” in , eds Pylkkänen P. Plykkö P. Hautamäki A. (Amsterdam: IOS Press).

[B110] Quinto-Pozos D. (2007). Can constructed action be considered obligatory. 117 1285–1314. 10.1016/j.lingua.2005.12.003

[B111] Sams J. (2010). Quoting the unspoken: an analysis of quotations in spoken discourse. 42 3147–3160. 10.1016/j.pragma.2010.04.024

[B112] Schembri A. Jones C. Burnham D. (2005). Comparing action gestures and classifier verbs of motion: evidence from Australian Sign Language, Taiwan Sign Language, and nonsigners’ gestures without speech. 10 272–290. 10.1093/deafed/eni029 15858072

[B113] Sherzer J. (1972). Verbal and nonverbal deixis: the pointed lip gesture among the San Blas Cuna. 2 117–131. 10.1017/S0047404500000087

[B114] Sherzer J. (1991). The Brazilian thumbs-up gesture. 1 189–197. 10.1525/jlin.1991.1.2.189

[B115] Sidnell J. (2006). Coordinating gesture, talk, and gaze in reenactments. 39 377–409. 10.1207/s15327973rlsi3904_2

[B116] Slama-Cazacu T. (1973). Berlin: Mouton de Gruyter 10.1515/9783110800821

[B117] Sperber D. Wilson D. (1986). Oxford: Basil Blackwell.

[B118] Streeck J. (2009). Amsterdam: John Benjamins 10.1075/gs.2

[B119] Supalla T. (1978). “Morphology of verbs of location and motion in American Sign Language,” in , eds Caccamise C. Hicks D. (Coronado, CA: National Association of the Deaf), 27–46.

[B120] Supalla T. (2003). “Revisiting visual analogy in ASL classifier predicates,” in , ed. Emmorey K. (Mahwah, NJ: Lawrence Erlbaum Associates), 249–257.

[B121] Sutton-Spence R. Woll B. (1999). Cambridge: Cambridge University Press 10.1017/CBO9781139167048

[B122] Tannen D. (1989). Cambridge: Cambridge University Press.

[B123] Thompson S. A. Couper-Kuhlen E. (2005). The clause as a locus of grammar and interaction. 7 81–505. 10.1177/1461445605054403

[B124] Tomasello M. (2003). Cambridge, MA: Harvard University Press.

[B125] Vigliocco G. Perniss P. Vinson D. (2014). Language as a multimodal phenomenon: implications for language learning, processing and evolution. 369:20130292. 10.1098/rstb.2013.0292 25092660 PMC4123671

[B126] Wilcox S. (2007). “Routes from gesture to language,” in , eds Pizzuto E. Pietrandrea P. Simone R. (Berlin: Mouton de Gruyter), 107–131.

[B127] Wilkins D. (2003). “‘Why pointing with the index finger is not a universal (in socio-cultural and semiotic terms)’,” in , ed. Kita S. (Mahwah, NJ: Lawrence Erlbaum Associates), 171–215.

[B128] Wilson D. Sperber D. (1993). Linguistic form and relevance. 90 1–25. 10.1016/0024-3841(93)90058-5

